# Effects of etibatide combined with emergency percutaneous coronary intervention on blood perfusion and cardiac function in patients with acute myocardial infarction

**DOI:** 10.12669/pjms.37.1.2950

**Published:** 2021

**Authors:** Ying Hu, Fan-xing Qi, Li-na Yu, Wei Geng

**Affiliations:** 1 Ying Hu, Department of Cardiology, Baoding First Central Hospital, Baoding 071000, Baoding, China; 2 Fan-xing Qi, Department of Neurology, Baoding First Central Hospital, Baoding 071000, Baoding, China; 3 Li-na Yu, Department of Cardiology, Baoding First Central Hospital, Baoding 071000, Baoding, China; 4 Wei Geng, Department of Cardiology, Baoding First Central Hospital, Baoding 071000, Baoding, China

**Keywords:** Acute myocardial infarction, Etibatide, Myocardial contrast echocardiography, Cardiac function

## Abstract

**Objectives:**

To investigate the effects of etibatide combined with emergency percutaneous coronary intervention (PCI) on blood perfusion and cardiac function in acute myocardial infarction (AMI) patients.

**Methods:**

This was a prospective, randomized, controlled study. From November 2015 to June 2019, 196 patients with ST-segment elevation myocardial infarction (STEMI) undergoing emergency PCI admitted to Baoding First Central Hospital were enrolled. The 196 STEMI patients were randomly divided into experimental group and control group. In the experimental group, STEMI patients were treated with emergency PCI + etibatide; while in the control group, only PCI was performed. Observation indexes included: general data, myocardial perfusion and cardiac function indexes and major adverse cardiac events (MACE).

**Results:**

There was no significant difference in general data between the two groups (*P* > 0.05). The rate of ST-segment resolution (STR) in the experimental group was better than that in the control group (*P* < 0.05). In myocardial contrast echocardiography (MCE), higher peak intensity (PI) and shorter time-to-peak (TP) were observed in the experimental group compared with the control group (*P* < 0.05). The platelet aggregation rate was compared between the two group at the time points of before PCI, after PCI and two hour after drug withdrawal, and there was no significant change in the platelet aggregation rate of the control group between different time points (before PCI, after PCI and two hour after drug withdrawal); while the platelet aggregation rate of the experimental group was significantly lower after PCI and two hour after drug withdrawal than that before PCI (*P* < 0.05), and an obviously decreased platelet aggregation rate was found in the experimental group(*P* < 0.05). After three months of follow-up, there was one case of MACE in the experimental group and 1 case of MACE in the control group, without any difference in the incidence of MACE between the two groups (*P* > 0.05).

**Conclusion:**

Etibatide combined with emergency PCI could improve myocardial reperfusion and cardiac function in patients with acute STEMI without increasing the incidence of MACE.

## INTRODUCTION

Acute Myocardial Infarction (AMI) was considered as a serious threat to the life and health of human beings. It has been suggested that treatment and prognosis of AMI were closely related to the time of diagnosis and treatment. Early opening of diseased vessels and rapid recovery of effective myocardial reperfusion were important measures to reduce the mortality and improve the prognosis of AMI patients.^1^

Many studies have found that the platelet glycoprotein (GP) IIb/IIIa receptor antagonist played a role in inhibiting platelet aggregation in the final link by inhibiting the binding of GP IIb/IIIa receptor to fibrinogen. Tirofiban, as a representative drug, has been widely used in recent years. However, it has also been found that there was an increased risk of bleeding with the application of tirofiban, and patients were clinically treated with tirofiban in small dose.^2^-^5^ With the development of another representative drug, etibatide, there was a doubt in the application of etibatide in continually maintaining good clinical outcomes without increasing the risk of bleeding, thus the effect of etibatide was still needed to be further studied. Myocardial contrast echocardiography (MCE), one of the new imaging techniques to detect the status of viable myocardium, could use special ultrasound imaging technology to evaluate the perfusion status of myocardial microcirculation in terms of capillaries on the basis of ordinary echocardiography, thereby evaluating the viable myocardium and predicting the potential of myocardial function recovery in the area.^6^ The study aimed to evaluate the effects of etibatide combined with emergency PCI on myocardial perfusion in AMI patients by MCE.

## METHODS

The study was approved by the institutional ethics committee of Baoding First Central Hospital, and the written informed consents were obtained from of all participants. This was a prospective, randomized, controlled study. From November 2015 to June 2019, 196 patients with ST segment elevation myocardial infarction (STEMI) undergoing emergency PCI admitted to the Baoding First Central Hospital. A random sequence software was used to generate 1-200 random sequence numbers.

Then, according to the order of patients’ visits, the random sequence numbers were taken in chronological order. Patients with the odd number were enrolled into the experimental group and patients with the even number into the control group. There were 100 cases in the experimental group, including 74 males and 26 females, with an average age of 59.2 ± 12.3 years old; and there were 96 cases in the control group, including 64 males and 32 females, with an average age of 61.3 ± 10.5 years old. There was no significant difference in gender and age between the two groups (*P* > 0.05). The sample size was calculated by a PASS software.

### Inclusion criteria

(1) age, 18-75 years old; and gender unlimited; (2) time of onset < 1two hourours, indications for emergency PCI, and no contraindication for antithrombotic drugs; and (3) written informed consent forms.

### Exclusion criteria

(1) the history of gastrointestinal hemorrhage, hematuria, etc. within 6 months before inclusion; (2) blood pressure > 180/110 mmHg (1 mmHg = 0.133 kPa); (3) hematological system diseases such as coagulation disorders, blood platelet disorder, severe anemia, etc.; (4) the history of hemorrhagic cerebrovascular disease within one year, known cerebrovascular malformation, cerebral aneurysm, etc.; (5) the presence of serious retinopathy (hemorrhage or exudation); (6) the history of surgery in the past six months, and cardiopulmonary resuscitation performed in the past 2 weeks; (7) aortic dissection; and (8) severe heart, lung, liver and kidney insufficiency.

### Treatment methods

In the experimental group, patients were performed with intracoronary injection of 180 μg/kg etibatide twice with an interval of 10 minutes after the guide wire was given intravenously to pass through the diseased vessel, and then 2 μg/kg·min was intravenously pumped for 36 hours. In the control group, patients received no injection or pumping of etibatide. STEMI patients chewed aspirin enteric-coated tablets (300 mg) and ticagrelor (180 mg) before coronary angiography (CA). After PCI, patients were given routine oral administration of aspirin enteric-coated tablets (100 mg, once in the morning), ticagrelor (90mg, twice a day), enoxaparin (100 u/kg, once /12h), nitrates, statins, sedatives and other drugs.

*CA* After the right radial (femoral) artery puncture was successfully carried out, a 6F arterial sheath was inserted, and heparin (3, 000 U) was injected. Then, a 5F TIG multi-functional angiography catheter was used for CA, and continuous ECG monitoring and blood pressure monitoring were conducted during the operation. If the patients needed further interventional therapy, heparin would be additionally added according to the standard of 100 U/kg during the operation. Patients in the experimental group were administered intracoronary injection of 180 μg/kg etibatide twice with an interval of 10 minutes, and then 2 μg/kg·min was intravenously pumped for 36 hours. At the same time, according to the degree of coronary artery stenosis, an appropriate balloon dilatation was selected, and stent placement was carried out. *MCE* Quantitative assessment of myocardial perfusion was directly carried out seven days after PCI by MCE with the use of EPIQ 7 ultrasound system.

### Observation indexes

1. clinical data: age, gender, underlying diseases (hypertension, diabetes mellitus, hyperlipidemia), smoking history, Killip classification, the number of stent implantation, the number of diseased vessels, etc. Creatine kinase (CK), creatine kinase-MB (CKMB) and troponin (TnI) were monitored every two hourours after admission, and peak values were recorded.
2. Indexes of myocardial perfusion and cardiac function: (1) the rate of 2h-ST segment resolution and postoperative thrombolysis in myocardial infarction (TIMI) blood flow classification; (2) the peak values of CK, CK-MB and TnI; and (3) MCE 7 days after operation: PI and TP. 3. Platelet aggregation rate: the platelet aggregation rated induced by adenosine diphosphate (ADP) were detected before operation, during operation and two hour after operation. 4. All patients were followed up for three months by outpatient and telephone, and major adverse cardiac events (MACE) were recorded, including stent thrombosis, post-infarction angina pectoris, acute myocardial reinfarction, cardiac insufficiency and sudden cardiac death.

### Statistical analysis

SPSS20.0 statistical software was used for data processing. The measurement data were expressed as mean ± standard deviation (x ± s), group t-test was used for comparisons between two groups, and paired t-test was used for comparisons before and after treatment. The enumeration data was expressed by percentage (%), and Chi-square test was used for comparisons. *P* < 0.05 indicated that the difference was statistically significant.

## RESULTS

There was no significant difference in gender, age, the percentage of underlying diseases and the number of diseased vessels between the two groups (*P* > 0.05) (Table-I).As regards changes of myocardial enzymogram and electrocardiogram: There was no significant difference in peak values of CK, CK-MB and TnI between the two groups (*P* > 0.05). The rate of ST segment resolution in the experimental group was higher than that in the control group (*P* < 0.05) (Table-II). There was no significant difference in the ratio of TIMI3 blood flow between the two groups (*P* > 0.05); after MCE, higher PI and lower TP were observed in the experimental group compared with the control group (*P* < 0.05) (Table-II).

**Table-I:** Comparisons of general information between the two groups.

Items	Experimental group (n=100)	Control group (n=96)	T/χ^ 2^	P
Age (years)	60.15 ± 11.89	61.91 ± 11.18	0.576	0.521
*Gender [n(%)]*				
Male	74(74.0)	32(33.3)	0.632	0.427
Female	26(26.0)	32(33.3)	0.465	0.377
Hypertension [n(%)]	64 (64.0)	58 (60.4)	0.712	0.501
Type 2 diabetes mellitus [n(%)]	26 (26.0)	20 (20.8)	0.364	0.546
Hyperlipoidemia [n(%)]	48 (48.0)	51 (53.1)	0.611	0.561
History of smoking [n(%)]	59 (59.0)	48 (50.0)	0.942	0.484
*The number of diseased vessels [n(%)]*				
1	62 (62.0)	56 (58.3)	0.418	0.569
2	20 (20.0)	24 (25.0)	0.352	0.533
3	18 (18.0)	16 (16.7)	0.021	0.932

Figures in this tale needs to be corrected.

**Table-II:** Comparisons of changes in electrocardiogram and myocardial enzymes and myocardial perfusion indexes between the two groups.

Items	Experimental group (n = 100)	Control group (n = 96)	T/χ^ 2^	P
ST resolution >70%	92 (92.0)	72 (75.0)	5.821	0.021
CK peak value (U/L)	1620.15 ± 301.89	1531.91 ± 310.18	-1.576	0.221
CK-MB peak value (U/L)	151.38 ± 28.27	149.11 ± 23.08	-0.876	0.421
TnI (ng/L)	19.74 ± 2.91	19.85 ± 2.89	0.041	0.821
TIMI3 grade [n(%)]	96 (96.0)	84 (87.5)	2.633	0.128
PI	6.26 ± 0.81	5.91 ± 0.83	-2.513	0.018
TP	14.15 ± 0.69	14.62 ± 0.65	3.508	0.001

Notes: CK, creatine kinase; CK-MB, creatine kinase-MB; TnI, troponin; TIMI, thrombolysis in myocardial infarction; PI, peak intensity; TP, time-to-peak.

The platelet aggregation rate was compared between the two group at the time points of before PCI, after PCI and two hour after drug withdrew, and the results found that there was no significant change in the platelet aggregation rate of the control group between different time points (before PCI, after PCI and two hour after drug withdrawal); while the platelet aggregation rate of the experimental group was significantly lower after PCI and two hour after drug withdrawal than that before PCI (*P* < 0.05), and an obviously decreased platelet aggregation rate was found in the experimental group compared with the control group at the same time points (*P* < 0.05) (Table-III). After three months of follow-up, there was one case of MACE in the experimental group and one case of MACE in the control group, but without significant difference in the incidence of MACE between the two groups (χ ^2^ = 0.001, *P* > 0.05).

**Table-III:** Comparisons of platelet aggregation rates (%) between the two groups.

Items	Experimental group (n = 100)	Control group (n = 96)	t	P
Before PCI	45.3 ± 4.7	45.6 ± 5.4	-0.415	0.679
After PCI	4.6 ± 1.9	47.9 ± 7.4	-56.608	0.000
two hour after drug withdraw	16.9 ± 6.7	46.4 ± 6.1	-32.193	0.000

Note: PCI, percutaneous coronary intervention.

## DISCUSSION

STEMI was one of the critical heart diseases endangering human life and health. There were increasing trends in terms of the incidence rate, the hospitalization rate and the mortality rate of coronary heart disease in the younger, especially for AMI.^7^,^8^ In recent years, the guidelines on AMI issued by American Heart Association/American College of Cardiology (ACC/AHA) and European Society of Cardiology (ESC) have been continuously developed and updated based on studies on early detection and diagnosis and early reperfusion therapy of AMI, and great progress has been made in the diagnosis and treatment of AMI.^9^ Currently, the guidelines have pointed out that emergency PCI was the preferred method to open the diseased vessels in STEMI patients and to realize revascularization.^10^ A previous study have demonstrated that the peak values of CK and CK-MB were positively correlated with the infarct size of STEMI patients^11^, which was consistent with our results, that is, higher peak values of CK and CK-MB were associated with larger the infarct size. It has been suggested the gold standard for successful reperfusion was to reach TIMI3 blood flow in the infarction-related vessels. However, there was no significant difference in TIMI3 blood flow ratio between the experimental group and the control group. Previous evidence revealed that the early resolution of ST segment in infarcted vessels-related leads was one of the indexes for myocardial reperfusion.^12^ Moreover, the rate of ST segment resolution was an important index of myocardial perfusion in patients with myocardial infarction after emergency PCI, and was a predictor of MACE within 12 months.^13^ Importantly, the rate of ST segment resolution < 70% indicated that there was no-reflow phenomenon of coronary arteries in patients.^14^ In accordance with foreign studies, the rate of ST segment resolution in the experimental group was higher than that in the control group, confirming that a small dose of etibatide could improve myocardial perfusion after emergency PCI and reduce the incidence of MACE in patients with acute STEMI. Some studies have shown that conventional PCI may lead to postoperative complications in different periods and degrees in patients with myocardial infarction, such as distal-end peripheral vessel microembolism, causing perioperative and postoperative long-term thrombotic events, one of the most common complications.^15^ It was considered that infarction-related vessels reaching TIMI3 blood flow was the gold standard of successful reperfusion. However, there was the failure of achieving effective myocardial tissue reperfusion in patients with diseased vessels reaching TIMI3 blood flow, accounting for 30%-50%, subsequently causing disturbance of myocardium metabolism, and finally resulting in obviously reduced treatment effect of emergency PCI.^16^ As suggested by an increasing number of studies, MCE could not only provide quantitative real-time analysis of myocardial perfusion, but also predict the prognosis of infarcted myocardium. Additionally, MCE could be used to evaluate myocardial microcirculation perfusion and changes of ischemic myocardium blood perfusion after coronary opening and reconstruction, and higher PI and shorter TP were suggested to be associated with better blood perfusion.^17^-^19^ The results of our study showed that TP of the experimental group were better than those of the control group, indicating that low dose of etibatide could improve myocardial perfusion.

The present study confirmed that the use of etibatide could quickly inhibit platelet aggregation and it had important clinical significance. The platelet aggregation rate measured immediately after PCI was significantly decreased relative to the baseline value in patients with the use of etibatide, and the significant decrease in the platelet aggregation rate lasted until two hour after drug withdrawal. However, there was no significant change of the platelet aggregation rate measured before PCT, immediately after PCI and two hour after PCI in the control group without the use of etibatide, suggesting that etibatide could inhibit platelet aggregation more rapidly and reduce thrombosis.^20^

Generally speaking, there was still a risk of short/long-term adverse cardiac events even after the diseased vessels were opened timely and effectively, such as arrhythmia, cardiac insufficiency and sudden death. Villanueva DLE et al. showed that the probability of developing MACE within one month was 5%-6% because no reflow event occurred during PCI.^21^ Myocardial perfusion disorder was closely related to the incidence of heart failure, infarct size and clinical prognosis, so that the prognosis of acute SEMI patients could be improved only by increasing the effective myocardial reperfusion. After three months of follow-up, we found that there was no increase in the incidence of bleeding complications and MACE in patients with the use of etibatide.

### Limitations of the study

(1) PCI was carried out together with a variety of other adjuvant treatments, which may interfere with the analysis of the therapeutic effect of etibatide; (2) the study had a small sample size, short follow-up period and no more detailed subgroup comparison. Our findings were still needed to be further confirmed by more in-depth studies in the future.

## CONCLUSION

The use of etibatide combined with emergency PCI could improve myocardial reperfusion and cardiac function in patients with acute STEMI, without increasing the incidence of MACE.

### Authors’ Contribution:

**YH and** **FXQ:** designed this study, prepared this manuscript and are responsible and accountable for the accuracy or integrity of the work.

**WG:** collected and analyzed clinical data.

**LNY:** significantly revised this manuscript.

**Ying Hu** and **Fan-xing Qi:** both considered as first author.

## References

[1] Zhai XB, Gu ZC, Liu XY (2019). Clinical pharmacist intervention reduces mortality in patients with acute myocardial infarction:A propensity score matched analysis. Eur J Hosp Pharm.

[2] Lev EI, Kornowski R, Teplisky I, Hasdai D, Rechavia E, Shor N (2005). The impact of adjunctive eptifibatide therapy with percutaneous coronary intervention for acute myocardial infarction. Int J Cardiovasc Intervent.

[3] O'Shea C, Tcheng JE (1999). Platelet glycoprotein IIb/IIIa integrin inhibition in acute myocardial infarction. J Invasive Cardiol.

[4] Kleiman NS, Tracy RP, Talley JD, Sigmon K, Joseph D, Topol EJ (2000). Inhibition of platelet aggregation with a glycoprotein IIb-IIIa antagonist does not prevent thrombin generation in patients undergoing thrombolysis for acute myocardial infarction. J Thromb Thrombolysis.

[5] O'Donoghue M, Antman EM, Braunwald E, Murphy SA, Steg PG, Finkelstein A (2009). The efficacy and safety of prasugrel with and without a glycoprotein IIb/IIIa inhibitor in patients with acute coronary syndromes undergoing percutaneous intervention:a TRITON-TIMI 38 (Trial to Assess Improvement in Therapeutic Outcomes by Optimizing Platelet Inhibition With Prasugrel-Thrombolysis In Myocardial Infarction 38) analysis. J Am Coll Cardiol.

[6] Min SY, Song JM, Shin Y, Sin MJ, Kim DH, Kang DH (2017). Quantitative segmental analysis of myocardial perfusion to differentiate stress cardiomyopathy from acute myocardial infarction:A myocardial contrast echocardiography study. Clin Cardiol.

[7] Hong JS, Kang HC (2015). Sex Differences in the Treatment and Outcome of Korean Patients with Acute Myocardial Infarction Using the Korean National Health Insurance Claims Database. Medicine (Baltimore).

[8] Normann J, Mueller M, Biener M, Vafaie M, Katus HA, Giannitsis E (2012). Effect of older age on diagnostic and prognostic performance of high-sensitivity troponin T in patients presenting to an emergency department. Am Heart J.

[9] Capodanno D, Alfonso F, Levine GN, Valgimigli M, Angiolillo DJ (2018). ACC/AHA Versus ESC Guidelines on Dual Antiplatelet Therapy:JACC Guideline Comparison. J Am Coll Cardiol.

[10] Cao B, Qu F, Liu X, Gao C, Fu Q, Jiang C (2019). Short-term efficacy of ticagrelor in acute ST-segment elevation myocardial infarction patients undergoing an emergency percutaneous coronary intervention. Aging (Albany NY).

[11] Mukherjee P, Jain M (2019). Effect of ischemic postconditioning during primary percutaneous coronary intervention for patients with ST-segment elevation myocardial infarction:A single-center cross-sectional study. Ann Card Anaesth.

[12] Engstrøm T, Kelbæk H, Helqvist S, Høfsten DE, Kløvgaard L, Clemmensen P (2017). Effect of Ischemic Postconditioning during Primary Percutaneous Coronary Intervention for Patients With ST-Segment Elevation Myocardial Infarction:A Randomized Clinical Trial. JAMA Cardiol.

[13] Reinstadler SJ, Baum A, Rommel KP, Eitel C, Desch S, Mende M (2015). ST-segment depression resolution predicts infarct size and reperfusion injury in ST-elevation myocardial infarction. Heart.

[14] Chettibi M, Benghezel S, Bertal S, Nedjar R, Bouraghda MA, Bouafia MT (2015). No reflow:quels facteurs prédictifs?[No reflow:What are the predictors?]. Ann Cardiol Angeiol (Paris).

[15] Steinhubl SR, Kastrati A, Berger PB (2007). Variation in the definitions of bleeding in clinical trials of patients with acute coronary syndromes and undergoing percutaneous coronary interventions and its impact on the apparent safety of antithrombotic drugs. Am Heart J.

[16] Iwakura K, Ito H, Okamura A, Kurotobi T, Koyama Y, Date M (2007). Comparison of two- versus three-dimensional myocardial contrast echocardiography for assessing subendocardial perfusion abnormality after percutaneous coronary intervention in patients with acute myocardial infarction. Am J Cardiol.

[17] Jin J, Wang H, Song YM, Li AM, Qin J, Yu XJ (2010). Clinical outcome and left ventricular remodeling in AMI patients with insufficient myocardial reperfusion after recanalization. Clin Invest Med.

[18] Olszowska M, Kostkiewicz M, Podolec P, Rubis P, Tracz W (2009). Assessment of resting perfusion defect in patients with acute myocardial infarction:comparison of myocardial contrast echocardiography with contrast-enhanced magnetic resonance imaging. Kardiol Pol.

[19] Dwivedi G, Janardhanan R, Hayat SA, Lim TK, Greaves K, Senior R (2010). Relationship between myocardial perfusion with myocardial contrast echocardiography and function early after acute myocardial infarction for the prediction of late recovery of function. Int J Cardiol.

[20] Jamasbi J, Ayabe K, Goto S, Nieswandt B, Peter K, Siess W (2017). Platelet receptors as therapeutic targets:Past present and future. Thromb Haemost.

[21] Villanueva DLE, Tiongson MD, Ramos JD, Llanes EJ (2020). Monocyte to High-Density Lipoprotein Ratio (MHR) as a predictor of mortality and Major Adverse Cardiovascular Events (MACE) among ST Elevation Myocardial Infarction (STEMI) patients undergoing primary percutaneous coronary intervention:a meta-analysis. Lipids Health Dis.

